# Noise-induced hearing loss: a 10-year analysis of notifications according to the Brazilian Classification of Occupations

**DOI:** 10.47626/1679-4435-2023-1163

**Published:** 2024-09-24

**Authors:** Maria Isabel Pereira de Rezende, Bruna Rafaela Cruz Barbosa, Arthur Henrique Gonçalves, Narciso Ferreira dos Santos Neto, Luiz Henrique Rodrigues Souza, Lucineia Pinho

**Affiliations:** 1 School of Medicine, Universidade Estadual de Montes Claros (UNIMONTES), Montes Claros, MG, Brazil; 2 Professional Master’s Degree in Computational Modeling and Systems, UNIMONTES, Montes Claros, MG, Brazil; 3 Graduate Program in Primary Health Care, UNIMONTES, Montes Claros, MG, Brazil

**Keywords:** occupational health, hearing loss, noise-induced, hearing loss, noise, occupational, saúde do trabalhador, perda auditiva provocada por ruído, perda auditiva, ruído ocupacional

## Abstract

**Introduction:**

Noise-induced hearing loss has a high incidence among Brazilian workers, causing socioeconomic and cognitive impairments.

**Objectives:**

To describe noise-induced hearing loss according to professional occupation between 2012 and 2021.

**Methods:**

This was a cross-sectional and descriptive study of Brazilian workers affected by noise-induced hearing loss. Data were collected from noise-induced hearing loss notifications filed with the Sistema de Informação de Agravos de Notificação in Brazil. The absolute and relative frequencies of sociodemographic variables and occupational characteristics were assessed.

**Results:**

A total of 7,413 cases of noise-induced hearing loss were notified in Brazil. Workers in industrial production were the most affected (56.4%). There was a higher prevalence in White (45.4%), male (87.3%) participants aged between 50 and 59 years (42.5%) from the Southeast region (43%). In this study, 3.5% of participants were temporarily removed from their position, 42.6% reported using personal protective equipment, and 65.7% did not use collective protective equipment. The most prevalent type of noise was continuous noise (33%).

**Conclusions:**

Noise-induced hearing loss is prevalent among Brazilian workers and varies according to each occupational group. Cases of noise-induced hearing loss are underreported in Brazil.

## Abstract (pt)

**Introdução:**

A perda auditiva induzida por ruído possui alta incidência entre os trabalhadores brasileiros, acarretando prejuízos socioeconômicos e cognitivos.

**Objetivos:**

Descrever a perda auditiva induzida por ruído no Brasil segundo a ocupação entre os anos de 2012 e 2021.

**Métodos:**

Tratou-se de um estudo transversal e descritivo, cuja população-alvo foi trabalhadores brasileiros afetados pela perda auditiva induzida por ruído. Os dados foram coletados a partir das fichas de notificação de perda auditiva induzida por ruído, oriundas do Sistema de Informação de Agravos de Notificação no período de 2012 a 2021. Foram analisadas as frequências absoluta e relativa de variáveis sociodemográficas e de características do trabalho.

**Resultados:**

Foram notificados 7.413 casos de perda auditiva induzida por ruído no país, sendo o grupo de trabalhadores da produção de bens e serviços industriais o mais afetado (56,4%). Houve maior proporção de casos em indivíduos do sexo masculino (87,3%), com cor de pele branca (45,4%), na faixa etária de 50 a 59 anos (42,5%) e da região Sudeste (43%). Entre os trabalhadores, 3,5% foram afastados temporariamente de suas ocupações, 42,6% faziam uso de medidas de proteção individual e 65,7% não utilizavam proteção coletiva. O ruído contínuo foi predominante (33%).

**Conclusões:**

A perda auditiva induzida por ruído é prevalente entre os trabalhadores brasileiros e varia conforme o grupo ocupacional. Os casos de perda auditiva induzida por ruído são subnotificados no Brasil.

## INTRODUCTION

Hearing is an essential tool for cognitive development, as it assists in the development of skills such as communication.^1^ In Brazil, the emergence of public policies focused on ear and hearing care was facilitated by the 1988 Constitution of the Federative Republic of Brazil. Law No. 8.080 of September 19, 1990, guarantees access to health care to people with hearing loss in the Brazilian Public Health System (SUS, Sistema Único de Saúde). In this setting, often marked by disregard for the psychological and social well-being of people with hearing loss, the National Policy for Hearing Care (PNASA, Política Nacional de Atenção à Saúde Auditiva) was developed in 2004, proposing actions for prevention and health promotion.^2^

Approximately 15% of the adult population in the world suffers from some degree of hearing loss, which can be categorized as mild, moderate, or severe.^3^ Hearing loss, whether partial or total, entails a series of psychological, social, and economic consequences.^4^ There are several factors that can lead to hearing loss, including socioeconomic factors, age, exposure time, type of noise, and especially professional occupation. The higher incidence of noise-induced hearing loss (NIHL) according to occupation appears to be multifactorial, associated both with social factors that predispose the individual to specific work environments and with conditions experienced during the working day.

In this sense, there is a need for a better understanding of work-related NIHL according to professional occupation. The level of hazardous noise in industries is inversely proportional to the technological development of the machinery, as more rudimentary forms of production predispose workers to greater exposure to noise arising from the production process.

Regulatory Standard No. 15, approved by the Brazilian Ministry of Labor Ordinance No. 3,214 of June 8, 1978,^5^ established acceptable noise levels (in dB) according to the maximum daily exposure allowed in hours. Because exposure is regulated according to time, the distribution of NIHL cases is not homogeneous across different occupations. Specific safety actions, as well as the use of personal protective equipment (PPE), must be established to protect workers from exposure to the noise levels of specific occupation.

Identifying the specific incidences, distributions, historical trends, and determinants of injuries can support the development of actions aimed at preventing, promoting, and protecting workers’ health.^6^ To this end, cases of NIHL are notified to the Information System for Notifiable Diseases (Sistema de Informação de Agravos para Notificação, SINAN).^7^ This system provides mechanisms for interpreting the causes of notifiable diseases, as it performs a dynamic analysis of the occurrence of an event in the population. Local health services that detect a relationship between hearing loss and continued exposure to noise in the occupational environment must complete the NIHL Investigation Form on SINAN, which is an important instrument for assessing the health of workers.

When considering the work environment, the worker must be seen as an integral part of the occupational process. Therefore, in addition to what is universally offered to SUS users, we must also focus on the different risks offered exclusively by the profession.

In this context, there is an urgent need to understand the distribution of NIHL cases in the workplace, especially according to different work groups, given the heterogeneity of aggravating factors for hearing health according to occupation. The objective of this study was to describe NIHL occurrence according to professional occupation between 2012 and 2021.

## METHODS

This was a cross-sectional and descriptive study using secondary data from compulsory NIHL notifications in Brazil according to professional occupation between 2012 and 2021.

According to data from the Brazilian Institute of Geography and Statistics (IBGE),^8^ Brazil has an estimated population of 215,521,552 inhabitants, of whom 79 million (46.7%) are economically active. The country has 27 federative units and 5,570 cities, with a total area of 8,514,876 km^2^. The Human Development Index (HDI) of Brazil is 0.754.

To assess NIHL occurrence, data relating to confirmed cases reported in SINAN^7^ in Brazil were used, according to occupation, between 2012 and 2021. SINAN is a nationwide database that provides epidemiological data, mostly originated from the notification and investigation of cases of diseases and conditions included in the national list of notifiable diseases.^9^

NIHL is a work-related notifiable disease registered in SINAN, as established by Consolidation Ordinance No. 4 of September 28, 2017 (4/2017).^9^ Notifications are recorded on an individual notification form, which is completed by patient care units for each patient suspected of having a notifiable disease or a disease of public health significance. This document must be sent to the services responsible for information and/or epidemiological surveillance at the Municipal Departments, which must store the files on magnetic media and forward them to the State Departments of Health on a weekly basis.

The data of interest for our study originated from secondary databases, available online and for free through the SUS Department of Informatics (DATASUS) via health information from the TABNET program.^10^ The codes from the Brazilian Classification of Occupations (CBO)^11^ were used to categorize NIHL according to the occupation of the affected individuals, aiming to identify the group with the highest incidence.

The NIHL assessment included the type of noise predominant in the work environment; the common treatment after the work accident; the reporting region; and the progress of the case. Other variables included year of notification (2012 to 2021) and sociodemographic and occupational characteristics. The sociodemographic characteristics analyzed were sex (male or female); age group (in years); race/skin color (White, Black, Mixed-race, Asian, Indigenous, or ignored/blank); and education (illiterate; incomplete 1st to 4th grade of elementary school; complete 4th grade of elementary school; incomplete 5th to 8th grade of elementary school; complete elementary education; complete and incomplete secondary education; complete and incomplete higher education; not applicable; or ignored/blank). The occupational characteristics analyzed were professional status (self-employed, cooperative member, or independent contractor, grouped into “self-employed”; registered employees, temporary workers, and public servants, grouped into “hired employees”; statutory civil servants, unemployed, retired, employer, unregistered employee, others, ignored/blank), occupational category according to the CBO,^11^ and economic activity according to the National Classification of Economic Activities,^12^ grouped by section.

The data were compiled in Microsoft Excel® spreadsheets and subsequently exported to SPSS version 18.0 for Windows. Data were analyzed using descriptive statistics, and absolute (n) and relative (%) frequencies were estimated.

## RESULTS

**Figure 1:**
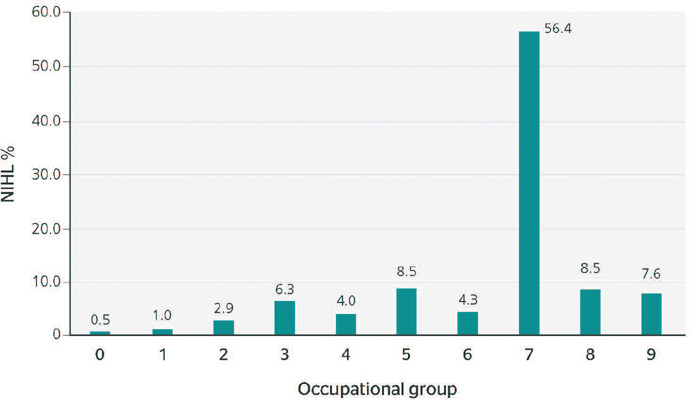
Distribution of the number of notifications of noise-induced hearing loss (NIHL) according to occupational group, Brazil, 2012 to 2021.

Between 2012 and 2021, 7,413 cases of NIHL were reported in Brazil. Figure 1 shows the number of NIHL notifications according to occupation. Group 7 represented 56.4% of all notifications in the country (n = 4,180), followed by Groups 5 (8.5%), 8 (8.5%), and 9 (7.6%).

0 = members of the armed forces, police, and military firefighters; 1 = members of the legislature, executive branch, or judiciary, senior public administration officials, senior managers of companies; 2 = scientists, artists, and similar workers; 3 = mid-level technicians; 4 = administrative workers; 5 = workers in commerce and similar workers; 6 = workers in agriculture, forestry, and fishery; 7 = workers in industrial production; 8 = workers in industrial production; 9 = workers in repair and maintenance services.

Group 7 (workers in industrial production [56.4%]) refers to occupational families in the industry who perform “artisanal” work, i.e., workers who know and work in all stages of the production process regardless of the use of simple or sophisticated equipment. The subgroups of Group 7 with the highest prevalence were those representing workers in the extractive industry and construction (n = 1,605; 38.40%), cross-functional workers (n = 1,266; 30.29%), and metal-processing workers (n = 780; 18.66%) (Chart 1).

**Chart 1:** Frequency of notifications of noise-induced hearing loss (NIHL) according to subgroups of Group 7 - workers in industrial production

Main subgroup	Occupation	n	%
71	Workers in the extractive industry and construction	1,605	38.40
78	Cross-functional workers	1,266	30.29
72	Metal-processing workers	780	18.66
76	Workers in the textile, tannery, clothing, and graphic arts industries	237	5.67
77	Workers in the wood and furniture industries	189	4.52
73	Electronic manufacturing and installation workers	54	1.29
75	Jewelers, glassmakers, ceramicists, and similar workers	46	1.10
74	Assemblers of precision and musical apparatus and instruments	2	0.05
79	Artisans	1	0.02
Group 7	Workers in industrial production	4,180	100.00

Table 1 shows the distribution of NIHL notifications according to sociodemographic characteristics, according to professional occupation between 2012 and 2021. As for sex, men were the most affected in all occupational groups, with prevalences above 50%. NIHL occurrence in Group 4 was the most equivalent regarding sex (men: 52.5% and women: 47.7%). Regarding education, there was a prevalence of individuals with complete secondary education in occupational Groups 0, 1, 3, 4, 5, 8, and 9. In Groups 6 and 7, there was a prevalence of participants with incomplete 5th to 8th grade of elementary school. Group 2 differs from the others, with a prevalence of people with complete higher education.

**Table 1:** Sociodemographic characteristics of notifications of noise-induced hearing loss (NIHL) according to occupation, Brazil, 2012 to 2021

Variables, n (%)	0	1	2	3	4	5	6	7	8	9	Total
Sex											
Female	0 (0.0)	10 (13.2)	85 (40.3)	119 (26.2)	143 (47.7)	215 (34.1)	18 (5.6)	284 (6.8)	51 (8.1)	18 (3.2)	943 (12.7)
Male	40 (100.0)	66 (86.8)	126 (59.7)	335 (73.8)	157 (52.5)	416 (65.9)	301 (94.4)	3,896 (93.2)	578 (91.9)	542 (96.8)	6,457 (87.3)
Age group, years											
20 to 29	0 (0.0)	2 (3.4)	9 (5.1)	23 (5.9)	46 (19.0)	30 (6.4)	10 (5.7)	156 (4.9)	28 (5.5)	24 (6.2)	328 (5.8)
30 to 39	7 (22.6)	7 (12.1)	38 (21.6)	58 (14.8)	56 (23.2)	82 (17.6)	23 (13.0)	579 (18.2)	85 (16.7)	68 (17.6)	1,003 (17.9)
40 to 49	14 (45.1)	28 (48.3)	70 (39.8)	142 (36.2)	79 (32.6)	145 (31.1)	45 (25.6)	1,088 (34.2)	169 (33.1)	122 (31.5)	1,902 (33.9)
50 to 59	10 (32.3)	21 (36.2)	59 (33.5)	169 (43.1)	61 (25.2)	209 (44.9)	98 (55.7)	1,357 (42.7)	228 (44.7)	173 (44.7)	2,385 (42.4)
Skin color											
Ignored/blank	17 (42.5)	19 (25)	36 (17)	98 (21)	70 (23.3)	127 (20.1)	42 (13.2)	964 (23.1)	186 (29.6)	152 (271)	1,711 (23.1)
White	14 (35)	42 (55.3)	120 (56.6)	209 (44.8)	123 (41)	269 (42.6)	201 (63)	1,851 (44.3)	286 (45.5)	254 (45.4)	3,369 (45.4)
Black	5 (12.5)	2 (2.6)	11 (5.2)	24 (5.2)	11 (3.7)	36 (5.7)	15 (4.7)	231 (5.5)	36 (5.7)	29 (5.2)	400 (5.4)
Asian	2 (5)	0 (0)	4 (1.9)	8 (1.7)	5 (1.7)	37 (5.9)	6 (1.9)	72 (1.7)	9 (1.4)	12 (2.1)	155 (2.1)
Mixed-race	2 (5)	13 (171)	41 (19.3)	126 (27)	90 (30)	157 (24.9)	52 (16.3)	1,054 (25.2)	111 (17.6)	113 (20.2)	1,759 (23.7)
Indigenous	0 (0)	0 (0)	0 (0)	1 (0.3)	1 (0.3)	5 (0.8)	3 (0.9)	8 (0.2)	1 (0.2)	0 (0)	19 (0.3)
Educational level											
Ignored/blank	24 (60)	39 (51.3)	60 (28.4)	161 (34.6)	107 (35.7)	244 (38.7)	113 (35.4)	1,509 (36.1)	279 (44.4)	248 (44.3)	2,784 (37.6)
Illiterate	0 (0.0)	0 (0.0)	1 (0.5)	0 (0.0)	1 (0.3)	10 (1.6)	6 (1.9)	26 (0.6)	5 (0.8)	2 (0.4)	51 (0.7)
Incomplete 1st to 4th grade of elementary school	1 (2.5)	1 (1.3)	1 (0.5)	15 (3.2)	9 (3)	36 (5.7)	37 (11.6)	407 (9.7)	51 (8.1)	41 (7.3)	599 (8)
Complete 4th grade of elementary school	1 (2.5)	3 (3.9)	5 (2.4)	8 (1.7)	8 (2.7)	34 (5.4)	41 (12.9)	386 (9.2)	33 (5.3)	34 (6)	553 (7, 5)
Incomplete 5th to 8th grade of elementary school	0 (0.0)	4 (5.3)	3 (1.4)	24 (5.2)	26 (8.7)	79 (12.5)	62 (19.4)	608 (14.6)	53 (8.4)	49 (8.8)	908 (12.3)
Complete elementary school	1 (2.5)	4 (5.3)	7 (3.3)	22 (4.7)	20 (6.7)	59 (9.3)	25 (7.8)	397 (9.5)	45 (7.2)	39 (7)	619 (8.4)
Incomplete secondary school	2 (5)	1 (1.3)	3 (1.4)	22 (4.7)	14 (4.6)	22 (3.5)	10 (3.1)	172 (4.1)	26 (4.1)	20 (3.6)	292 (3.9)
Complete secondary school	7 (17.5)	14 (18.4)	24 (11.4)	139 (29.8)	78 (26)	102 (16.2)	18 (5.6)	571 (13.7)	116 (18.5)	104 (18.6)	1,173 (15.8)
Incomplete superior education	1 (2.5)	0 (0.0)	6 (2.8)	22 (4.7)	20 (6.7)	15 (2.4)	1 (0.3)	25 (0.6)	4 (0.6)	7 (1.2)	101 (1.4)
Complete superior education	3 (7.5)	9 (11.9)	101 (47.9)	52 (11.2)	15 (5)	26 (4.1)	4 (1.4)	44 (1.1)	11 (1.7)	12 (2.1)	277 (3.7)
Not applicable	0 (0.0)	1 (1.3)	0 (0.0)	1 (0.2)	2 (0.6)	4 (0.6)	2 (0.6)	35 (0.8)	6 (0.9)	4 (0.7)	55 (0.7)

0 = members of the armed forces, police, and military firefighters; 1 = members of the legislature, executive branch, or judiciary, senior public administration officials, senior managers of companies; 2 = scientists, artists, and similar workers; 3 = mid-level technicians; 4 = administrative service workers; 5 = workers in commerce and similar workers; 6 = workers in agriculture, forestry, and fishery; 7 = workers in industrial production; 8 = workers in industrial production; 9 = workers in repair and maintenance services.

As age increased in Groups 3, 5, 6, 7, 8 and 9, there was also an increase in the number of accidents. In Groups 0, 1, 2, and 4, notifications were more prevalent in the 40 to 49 age group. The analysis of the total number of accidents according to age group showed that it increased according to advancing age. When analyzing the skin color of affected individuals, there was a prevalence of White people in all major occupational groups, ranging from 35% to 63%.

Regarding the occupational characteristics of individuals, we found that the Occupational Accident Report (CAT, Comunicação de Acidente de Trabalho) was not issued in 43.2% of notifications, and this prevalence ranged from 32.5% to 51.3% across occupational groups. Group 1 had the lowest CAT issuance rate, with only 2.5% of accidents being reported. Conversely, Group 8 presented the highest CAT issuance rate (23.8%). The use of collective protection equipment was identified in only 8% of the notifications. Group 9 had the highest prevalence of collective equipment use (13.2%), while Group 6 had the lowest prevalence (3.4%).

We observed that 42.6% of workers used PPE, while 35.6% did not. The subgroup that least used this type of protection was subgroup 5, with 45.6% of workers reporting not using PPE. The group that most used this equipment was Group 3, with 53.9% of workers reporting they used PPE. Of those who suffered an accident, 3.5% were placed on leave and 61.5% continued to work despite having suffered an accident.

Among the NIHL cases analyzed, continuous noise was the most prevalent type of noise, accounting for 33% of cases. Continuous noises were found to be more prevalent in Groups 1 (42%), 3 (37%), 4 (33%), 6 (36%), 8 (49%), and 9 (35%), while intermittent noises were only more prevalent in Group 2 (31%). Both types of noises were predominant in Groups 0 (35%), 5 (39%), and 7 (32%).

In terms of the region of residence, the Southeast region was found to have the highest recurrence of NIHL notifications, followed by the Central-West region. The Central-West region had the highest prevalence of NIHL notifications in Groups 2 (43%) and 5 (56%), while the South region was only predominant in Group 6 (51%). The Southeast region had the highest incidence of NIHL notifications for all other occupational groups.

## DISCUSSION

This study examined the notification of NIHL cases from 2012 to 2021. Occupational Group 7 (workers in industrial production) had the highest number of NIHL notifications.

Hearing loss is reported as the most prevalent sensory disability worldwide.^13^ More than half of the NIHL notifications belonged to occupational Group 7. One explanation for the high prevalence of NIHL notifications in Group 7 is that it is the second largest occupational group, with approximately 180 million workers.^11^ The prevalence of NIHL among industrial workers is in accordance with the findings by Shi et al.,^13^ who observed a hearing loss prevalence of approximately 50% among 23,261 noise-exposed workers in the mining and manufacturing industries. This highlights the environmental noise caused by machinery, equipment, and industrial processes,^14^ which affects the quality of life of Group 7 workers, and sheds light on the predominance of notifications.

Within occupational Group 7, subgroups 71 (workers in the extractive industry and construction) and 78 (cross-sectional workers) are particularly affected by NIHL, accounting for over half of NIHL notifications in Group 7. Fernandes & Carvalho^15^ examined the medical records of 375 construction workers who underwent occupational examinations in 2010 from a company in the Campinas metropolitan region, in the state of São Paulo, Brazil. They observed a prevalence of total or partial NIHL of 34.9%,^15^ which corroborates our findings.

Our study revealed a low prevalence of NIHL in Group 0, which comprises members of the armed forces, police officers, and military firefighters. This is in alignment with the findings of Masterson et al.,^16^ who conducted a study on NIHL prevalence across various occupational sectors between 2003 and 2012. Their study found that public safety professionals had the lowest prevalence of NIHL (approximately 7%) among the groups investigated. One possible explanation is the consistent use of PPE during training sessions that involve exposure to noise, a common characteristic of their occupational duties.^17^ The use of PPE is in line with the recommendations of NR-6.^18^

According to data on NIHL notifications by age group, the incidence of cases increases among professional groups as age increases. The prevalence of notifications among individuals aged 20-29 was lower compared with subgroups of individuals over 50 years old, further supporting the observation that NIHL increases with age. A comparison can be drawn between the current study and the research conducted by Gonçalves et al.,^19^ as both studies address the combined effects of age-related hearing loss and noise exposure on hearing health. Length of employment can also play a significant role in hearing loss.^13^

Continuous noise was found to be more prevalent in most of the professional groups analyzed compared with intermittent noise or exposure to both types of noise. Continuous noise is defined as sound with a sound pressure level (SPL) variation of up to 3 dB over an observation period greater than 15 minutes. Intermittent noise is defined as sound with a SPL variation of 3 dB or more over observation periods shorter than 15 minutes and longer than 0.2 seconds.^20^ Elevated levels of occupational noise are indeed a cause for concern due to their detrimental impact on the hearing health and overall well-being of workers. Tsimpida et al.^1^ assert that beyond the physical risk associated with hearing loss, there are also psychological consequences, such as anger and stress, which can lead to permanent sequelae for workers.

Regarding the region of residence, a higher prevalence of NIHL notifications was observed in the Southeast region. This data may be linked to the economic and industrial representativeness of the region, considering the prominent positions of the states of São Paulo and Minas Gerais in the national ranking of industrial social security contribution collection in 2017.^21^

There was a higher prevalence of NIHL notifications among men in all occupational categories. This is consistent with findings from Passos & Machado,^22^ who attributed the observed sex disparity to a lower participation of women in the work force compared with men, despite advancements in female education.

The analysis of skin color data indicates that the majority of the workers is White. There was a low prevalence of Indigenous, Yellow, and Black people in the total number of notifications. Several variables can explain this result, such as education, work experience, and the influence of racial discrimination that permeates social and labor market relations,^23^ as well as historical problems in the Brazilian professional field, such as racism in the labor market and the filling of a large part of the country’s precarious occupations by Black people and women.^24^

The use of collective protective equipment is low across all groups; in general, this means of protection is underutilized. Low prevalence may be related to high technological implantation costs for collective protective measures.^25^

Regarding the use of PPE, Group 3 (mid-level technicians) stands out for its high compliance rate. In contrast, Group 4 (administrative service workers) had the lowest percentage of PPE use. Regulatory Standard No. 6^18^ regulates the use of PPE in the workplace and establishes the obligations of employers. The failure to use PPE in activities with noises is considered irregular. Workers’ awareness of the risk of noise exposure is crucial, as it plays a crucial role in their proactive approach to noise prevention and adherence to hearing protection equipment use.^26^

There is a low rate of CAT issuance across all occupational groups. In Brazil, up until 2004, NIHL information was obtained from the Ministry of Social Security through CATs. Currently, the Ministry of Health, through Consolidation Ordinance No. 4/2017, has established technical procedures for the mandatory reporting of work-related health conditions in the SUS. NIHL is included in the national list of diseases and conditions, present in Consolidation Ordinance No. 4/2017, to be monitored through the sentinel unit surveillance strategy and its guidelines.

More than half of workers do not take time off work for treatment, which can be considered a problem. In cases of hearing loss, there is a low chance of improvement, even after the worker has been removed from the work environment. This information is consistent with the findings of the Ministry of Health’s NIHL Protocol, which states that hearing loss is irreversible and progressive, worsening over time with continued exposure, and that there is no effective treatment available.^4^

Our findings facilitate the understanding of the prevalence of NIHL by occupational group. The contributions of these findings include the development of actions aimed at protecting worker’s health and the identification of the most affected groups, allowing for the rational allocation of public resources to target the most vulnerable professional groups. Multiple actions could be implemented to address NIHL, including government-imposed penalties for noncompliant companies, a reporting hotline to increase notifications, enhanced legal support for sick leave, mandatory hearing aid provision for affected individuals, and increased awareness campaigns about hearing loss on social media. Occupational safety in places of employment is crucial for minimizing harm to workers’ health.

This study promotes worker health by raising awareness, providing a historical analysis, and highlighting the impact of occupation activities on hearing loss. The contributions of this study can promote actions aimed at improving the work environment, quality of life, and health of workers.

Some study limitations include the underreporting of NIHL cases in Brazil and the paucity of studies on workers’ health related to NIHL, particularly in occupational analyses. This underreporting and lack of studies impact the visibility of the disease and the development of actions for the prevention and promotion of workers’ health.^27^

## CONCLUSIONS

NIHL is prevalent among Brazilian workers and varies by occupational group. Group 7 (workers in industrial production) was the most affected group by NIHL. Our study highlights the need for increased reporting of NIHL cases in SINAN.^7^ This would provide a stronger foundation for actions to promote the health of workers, contribute to improved workplace safety, and enhance the quality of life for this segment of society.
